# *Purple Acid Phosphatase5* is required for maintaining basal resistance against *Pseudomonas syringae* in Arabidopsis

**DOI:** 10.1186/1471-2229-13-107

**Published:** 2013-07-29

**Authors:** Sridhar Ravichandran, Sophia L Stone, Bernhard Benkel, Balakrishnan Prithiviraj

**Affiliations:** 1 Department of Environmental Sciences, Faculty of Agriculture, Dalhousie University, Truro, NS B2N 5E3, Canada; 2 Department of Biology, Dalhousie University, Halifax, NS B3H 4J1, Canada; 3 Department of Plant and Animal Sciences, Faculty of Agriculture, Dalhousie University, Truro, NS B2N 5E3, Canada

**Keywords:** *Arabidopsis*, Plant defense responses, *PAP5*, *Pseudomonas syringae*, Phosphate starvation

## Abstract

**Background:**

Plants have evolved an array of constitutive and inducible defense strategies to restrict pathogen ingress. However, some pathogens still manage to invade plants and impair growth and productivity. Previous studies have revealed several key regulators of defense responses, and efforts have been made to use this information to develop disease resistant crop plants. These efforts are often hampered by the complexity of defense signaling pathways. To further elucidate the complexity of defense responses, we screened a population of T-DNA mutants in Colombia-0 background that displayed altered defense responses to virulent *Pseudomonas syringae* pv. *tomato* DC3000 (*Pst* DC3000).

**Results:**

In this study, we demonstrated that the Arabidopsis *Purple Acid Phosphatse5* (*PAP5*) gene, induced under prolonged phosphate (Pi) starvation, is required for maintaining basal resistance to certain pathogens. The expression of *PAP5* was distinctly induced only under prolonged Pi starvation and during the early stage of *Pst* DC3000 infection (6 h.p.i). T-DNA tagged mutant *pap5* displayed enhanced susceptibility to the virulent bacterial pathogen *Pst* DC3000. The *pap5* mutation greatly reduced the expression of pathogen inducible gene *PR1* compared to wild-type plants. Similarly, other defense related genes including *ICS1* and *PDF1.2* were impaired in *pap5* plants. Moreover, application of BTH (an analog of SA) restored *PR1* expression in *pap5* plants.

**Conclusion:**

Taken together, our results demonstrate the requirement of PAP5 for maintaining basal resistance against *Pst* DC3000. Furthermore, our results provide evidence that PAP5 acts upstream of SA accumulation to regulate the expression of other defense responsive genes. We also provide the first experimental evidence indicating the role *PAP5* in plant defense responses.

## Background

Plants are continuously exposed to a diverse array of microorganisms including beneficial mutualists, commensals, and pathogens. To defend against pathogens, plants have evolved an innate immune system to recognize and limit infection (reviewed in [1,2]). Activation of defense responses involves the initial recognition of pathogens by chemical cues (elicitors) or Pathogen Associated Molecular Patterns (PAMPs) that include bacterial lipopolysaccharides, flagellin, fungal chitin and ergosterol [3,4]. Recognition of PAMP by specific Pattern Recognition Receptors (PRRs) in the plasma membrane leads to activation of defense responses in both non-host and basal disease resistance [5]. Activation of PRRs subsequently induces the calcium-dependent protein kinase (CDPK) and mitogen-activated protein kinase (MAPK) signaling pathways leading to rapid ion fluxes, followed by transcriptional activation of defense responsive genes and synthesis of antimicrobial compounds to restrict infection [6,7].

Primarily, regulation of plant defense responses is mediated through the phytohormones salicylic acid (SA), jasmonic acid (JA) and ethylene (ET) [8,9]. However, in recent years other phytohormones including abscisic acid (ABA), auxins, gibberellins (GA), cytokines (CK) and brassinosteriods (BR) have been shown to mediate specific plant defense responses (reviewed in [2,10]). As plants are exposed to an array of pathogens with diverse infection strategies, activation of appropriate, pathogen-specific defense responses is vital for plant growth and productivity [11].

Plant pathogens are classified as biotrophs, necrotrophs or hemi-biotrophs based on their life style and infection strategy. Biotrophic pathogens live as obligate parasites that derive nutrients from living host tissues, while necrotrophs feed on dead tissues. Hemi-biotrophs behave as both biotroph and necrotroph depending on the stage of their life cycle [11]. Defense against biotrophs involves SA-dependent responses whereas necrotroph resistance is SA-independent relaying primarily on JA/ET-dependent pathways [9]. The SA signaling pathway is associated with transcriptional activation of pathogenesis related (PR) genes and the establishment of systemic acquired resistance (SAR) to provide enhanced, long lasting resistance to secondary infections [12,13]. By contrast, JA/ET signaling pathways are associated with resistance against necrotrophic pathogens and rhizobacteria-mediated induced systemic resistance (ISR), and are not typically associated with PR gene expression [12,14]. However, there are complex signaling and cross talk between the SA-dependent and SA-independent pathways [13].

Genetic screening of mutant plant populations has proved very useful for the functional analysis of defense responses [15-17]. In Arabidopsis, genetic screening has revealed a large number of mutants that exhibit altered responses to SA, JA and/or ET and are more susceptible to virulent pathogens [18]. Identification and characterization of enhanced disease susceptibility (*eds*) mutants, including a series of phytoalexin deficient (*pad*) mutants, have helped to elucidate a number of defense signaling pathways involved in both basal and induced defense responses [19-21].

Purple Acid Phosphatases (PAPs) belong to a family of binuclear metalloenzymes that exhibit diverse biological functions in plants, animals and bacterial species [22,23]. While the predominant role of PAPs in plants is regulation of Pi uptake, PAPs also contribute to other biological functions including peroxidation [24], ascorbate recycling [25], mediation of salt tolerance [26] and regulation of cell wall carbohydrate biosynthesis [27]. Plant PAPs share significant sequence similarity with mammalian tartarate-resistant acid phosphatases (TRAPs), which are involved in bone resorption [28], iron transport [29] and also in the generation of reactive oxygen species for microbial killing [30]. In humans, TRAP expression is restricted to activated macrophages where it aids in the generation of free radicals to enhance microbial killing [31]. Although numerous reports have emphasized the importance of PAPs in Pi acquisition, it has been difficult to assign a general physiological role to PAPs due to their diversity [32]. The Arabidopsis genome contains 29 *PAP* encoding genes [33]. Changes in *PAP* gene expression differs in response to Pi concentration where PAP11 and PAP12 are transcriptionally induced while PAP7-PAP10 and *PAP13* remain unchanged in response to Pi deprivation [33]. Kaffarnik and colleagues first reported the accumulation of PAP10 and a decrease in the abundance of PAP14 in the secretome of Arabidopsis cell culture following *P. syringae* infection, suggesting a role for PAPs in the host defense response [34]. Recently, Li *et al*., (2012) also provided the evidence that some soybean PAPs (GmPAPs) are involved in symbiosis under Pi starved conditions. PAPs carry predicted signal peptides and presumably are secreted, however the biological function of these proteins in the extracellular space is unknown [34].

Here we provide evidence that the Arabidopsis *PAP5* is involved in basal resistance against certain plant pathogens. *PAP5* mutant plants exhibited enhanced susceptibility to virulent isolate of *Pseudomonas syringae* pv. *tomato* DC3000. In addition, expression of defense related genes following *Pst* DC3000 infection were impaired in *pap5* plants.

## Results

### Identification of mutants exhibiting altered defense responses

**Figure 1:**
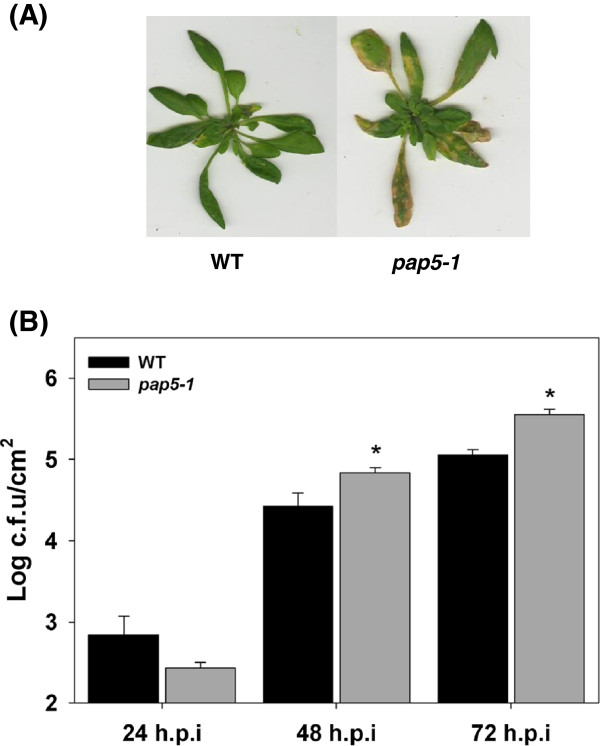
***pap5-1 *plants exhibit enhanced susceptibility to *Pst *DC3000. A**, Phenotype of *pap5-1* plants exhibiting extensive chlorosis and enhanced susceptibility to *Pst* DC3000. Plants were spray inoculated with 10^8^ c.f.u ml^–l^ and photographed after 5 days of inoculation. **B**, Growth of virulent *Pst* DC3000 in wild type (Col-0) and *pap5-1* leaves. Plants were spray inoculated with *Pst* DC3000 (10^8^ c.f.u ml^–l^) and bacterial growth in plant apoplast was determined as described in the materials and methods. The bars represent the mean and standard deviation from values of six to eight replicate samples. The experiment was repeated three times with similar results. An asterisk indicates significance (Student’s *t*-test; *P* < 0.05).

One thousand two hundred unique *Arabidopsis thaliana* (ecotype Col-0) T-DNA insertion lines were spray inoculated with the virulent isolate of *Pseudomonas syringae* pv. *tomato* DC3000 (*Pst* DC3000) and monitored for altered responses to the pathogen. Mutants exhibiting extensive chlorosis in comparison to wild-type plants, scored by visual examination, were designated as susceptible. Mutants exhibiting reduced chlorosis compared to wild type (Col-0) were designated resistant to *Pst* DC3000. T-DNA insertion lines were also tested for altered root colonization with the plant growth promoting rhizobacterial isolate *Pseudomonas putida* WCS358. Selected T-DNA lines were retested for response to *Pst* DC3000. A total of 24 T-DNA insertion lines exhibited either altered disease susceptibility, root colonization or both compared to wild-type plants (data not shown). The mutant line salk_126152C (*pap5-1*), which exhibited enhanced susceptibility to *Pst* DC3000 with extensive chlorosis on leaf tissues, was selected for further analysis (Figure 1A). Salk_126152C carried a T-DNA insertion in the gene coding for *Purple Acid Phosphatase5* (*PAP5*; At1G52940) (Genome-Wide Insertional Mutagenesis of *Arabidopsis thaliana*, 2003). The enhanced susceptibility phenotype of *pap5-1* plants was confirmed by assessing bacterial growth in leaf tissues post inoculation. As shown in Figure 1B, *pap5-1* plants had greater titers of bacteria at 48 and 72 hours post inoculation (h.p.i) compared to the wild-type plant. To ensure that the altered responses to the pathogen were caused by disruption of the *PAP5* gene and not by an unlinked mutation, a second knockout mutant line salk_081481C (*pap5-2*), carrying a T-DNA insertion on *PAP5* (At1g52940), was tested. *pap5-2* plants also exhibited the extensive chlorosis and higher titer of bacteria similar to that of in *pap5-1* plants (Additional file 1: Figure S1).

### Further characterization of *pap5-1* mutant plants

**Figure 2:**
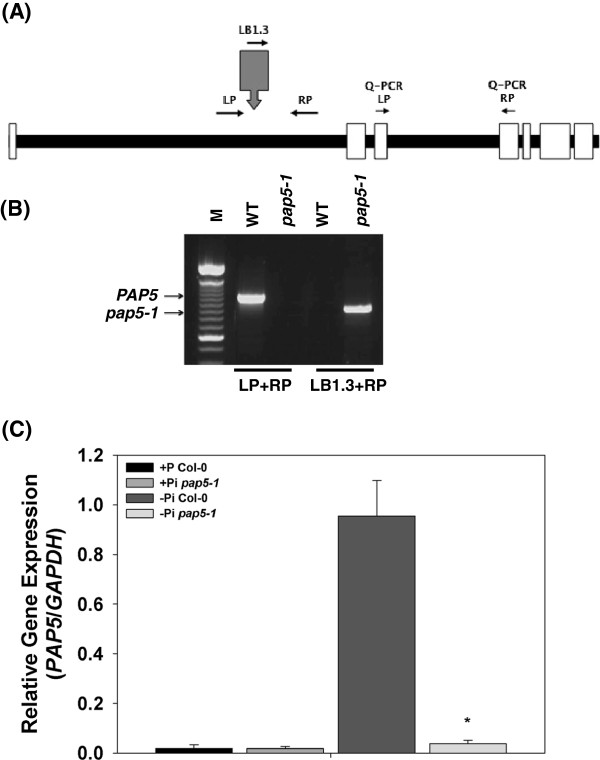
**Validation of T-DNA insertion in *pap5-1 *plants. A**, Schematic representation of *AtPAP5* (At1G52940); white boxes and solid lines represent exons and introns. T-DNA insertion is represented with a grey arrow and the solid arrows represent the primers used for genotyping and quantitative RT-qPCR. **B**, Location of the T-DNA insertion and homozygosity of *pap5-1* was confirmed by PCR using the gDNA from wild-type and *pap5-1* plants (M, 100 bp marker). A 30 cycle PCR reactions was performed with the primer pairs indicated. **C**, Relative expression of *PAP5* transcripts in response to prolonged Pi starvation; For prolonged Pi starvation wild type and *pap5-1* seedlings were germinated and grown in 0.5X MS media containing reduced Pi (0.25 mM). After seven days the seedlings were washed with sterile water and transferred to 0.5X MS with no Pi (0 mM). After 9 days the seedlings were harvested for gene expression analysis. Total RNA was extracted from wild-type and *pap5-1* plants as described in Methods. Transcript levels of *PAP5* was normalized to the expression of *GAPDH* in the same samples and expressed relative to the normalized transcript levels of Pi starved wild-type plants. The bars represent the mean and standard deviation from two independent experiments. Asterisks represents data sets significantly different from the wild-type data sets (*P* < 0.05 using one-tailed Student’s *t*-test).

Genotyping via polymerase chain reaction (PCR) confirmed that *pap5-1* (salk_126152C) carries a T-DNA insertion within the first intron (Figure 2A and 2B). To determine the impact of T-DNA insertion on transcript levels of *PAP5*, Reverse Transcription-quantitative PCR (RT-qPCR) was performed using gene specific primers (Figure 2A). Most PAPs are reported to be highly inducible under phosphate starvation (Pi). In our experiments, we did not observe an induction of *PAP5* in wild-type seedlings grown in the presence of phosphate (1.25 mM) or under phosphate starved conditions for 5 days (-Pi, 0 mM) (data not shown). We also observed that the expression of *PAP5* under optimal growing conditions was very low and this was confirmed with *PAP5* expression profile in the comprehensive microarray site https://www.genevestigator.com/gv/ (Additional file 2: Figure S2). Interestingly, we observed a marked increase in the expression *PAP5* when wild type seedlings were grown under prolonged phosphate starvation (Figure 2C). For prolonged Pi starvation wild-type seedlings were germinated in media containing reduced Pi (0.25 mM) for seven days and then transferred to media with no Pi (0 mM). After 9 days the seedlings were harvested for gene expression analysis. RT-qPCR analysis revealed a ~30 fold increase in transcript levels of *PAP5* in wild-type seedlings grown under prolonged phosphate starvation (-Pi) compared to seedlings grown in the presence of phosphate (+Pi) (Figure 2C). The expression of *PAP5* was not induced in both *pap5-1* (Figure 2C) and *pap5-2* (Additional file 3: Figure S3B) seedlings grown under prolonged phosphate starvation (-Pi). We did not observe any major alteration in germination, growth and development of *pap5* mutant plants compared to wild-type under optimal growth conditions (data not shown).

### Mutation in *PAP5* alters expression of host defense responsive genes and ROS production

**Figure 3:**
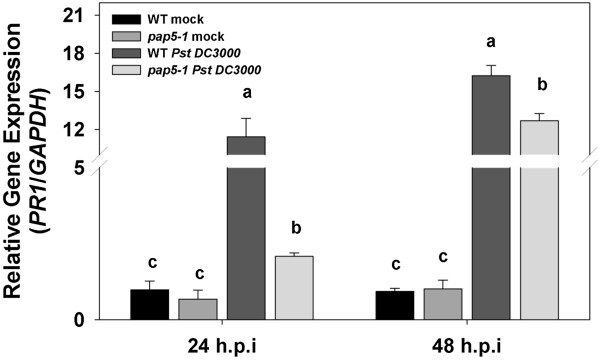
**Expression of *PR1 *in wild-type and *pap5-1 *plants after *Pst *DC3000 infection.** Transcript levels of *PR1* in wild-type and *pap5-1* plants were quantified after spray inoculation with virulent *Pst* DC3000 (10^8^ c.f.u ml^–l^). Total RNA was extracted from leaf tissues sampled 24 and 48 h.p.i*.* Transcript levels were normalized to the expression of *GAPDH* in the same samples. The transcript levels were expressed relative to the normalized transcript levels of mock infected wild-type plants. The bars represent the mean and standard deviation from two independent experiments. Significant differences (P < 0.05) are indicated by different letters.

**Figure 4:**
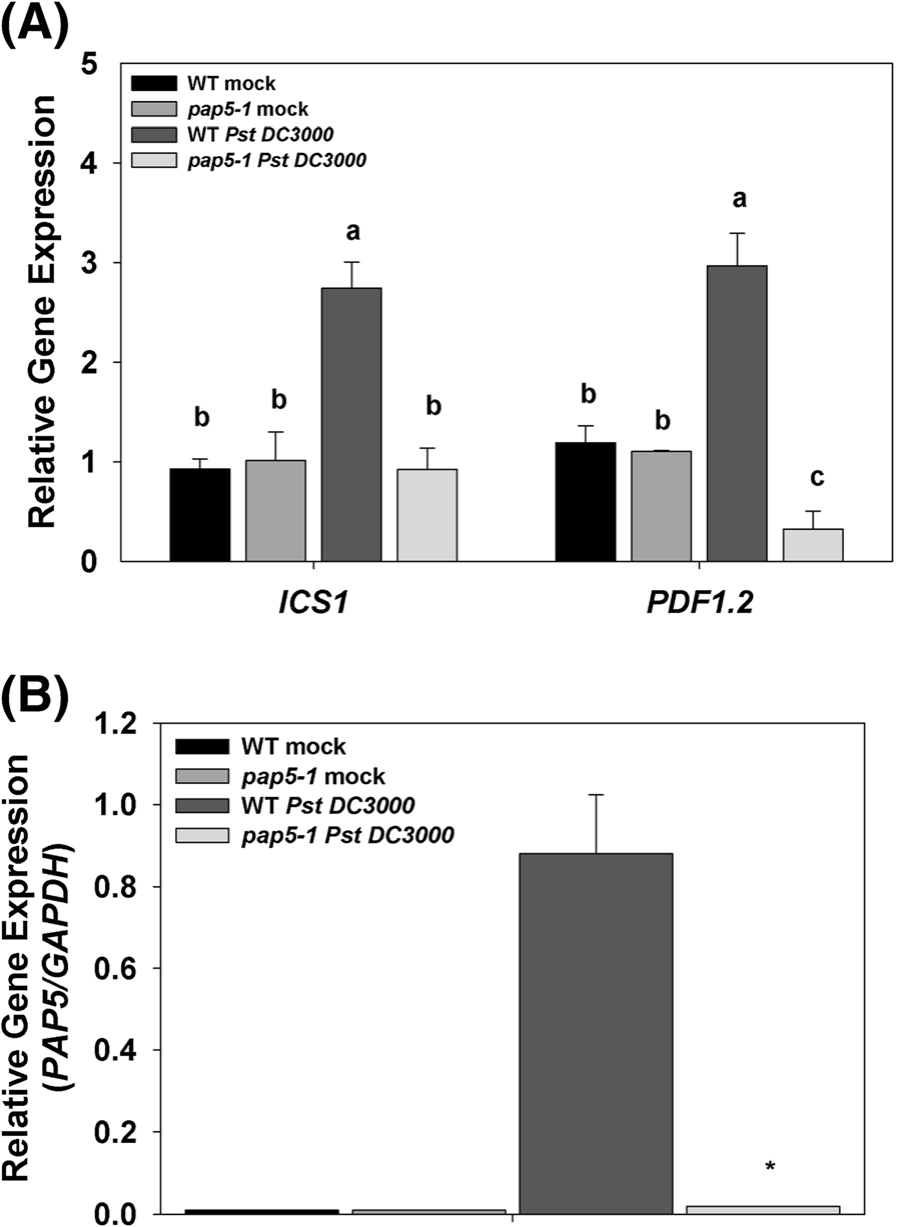
**Expression of *ICS1, PDF1.2 *and *PAP5 *in wild-type and *pap5-1 *plants after *Pst *DC3000 infection.** Transcript levels of *ICS1, PDF1.2* and *PAP5* in wild-type and *pap5-1* plants were quantified after spray inoculation with virulent *Pst* DC3000 (10^8^ c.f.u ml ^–l^). **A**, Expression *ICS1* and *PDF1.2* following *Pst* DC3000 infection. Total RNA was extracted from leaf tissues sampled at 24 h.p.i. Transcript levels were normalized to the expression of *GAPDH* in the same samples. The transcript levels were expressed relative to mock infected wild-type plants. **B**, Expression of *PAP5* following *Pst* DC3000 infection. Total RNA was extracted from leaf tissues 6 h.p.i. Transcript levels were normalized to the expression of *GAPDH* in the same samples and expressed relative to transcript levels of infected wild-type plants. The bars represent the mean and standard deviation from two independent experiments. Significant differences (P<0.05) are indicated by different letters. Asterisks indicate significant difference in transcript levels compared to wild-type (Students *t*-test; *P<0.05*).

To explore the enhanced susceptibility of *pap5-1* plants and to determine the role of *PAP5* in host defense responses, plants were spray inoculated with virulent isolate of *Pst* DC3000 (10^8^ c.f.u ml^–l^) and the transcript abundances of selected defense responsive genes, including the pathogenesis-related gene1 (*PR1*)*,* were determined. Infection of wild-type plants with the virulent isolate *Pst* DC3000 resulted in ~10-fold induction of the *PR1* transcript 24 h.p.i, while an increase of only ~2-fold was observed in *pap5-1* plants (Figure 3). The level of *PR1* transcripts in *pap5-1* plants following *Pst* DC3000 infection was variable at 48 h.p.i. However, the expression of *PR1* was a still less induced in *pap5-1* plants compared to wild-type (Figure 3). Expression of *isochorismate synthase1* (*ICS1*) was induced in wild-type plants (~2-fold) while no increase in transcript levels was observed in *pap5-1* plants*.* Although, expression of *plant defensin1.2* (*PDF1.2*) was induced (~2-fold higher) in wild-type plants, expression of *PDF1.2* was suppressed in *pap5-1* plants (Figure 4A). The expression pattern of these pathogenesis related genes were also confirmed using *Actin* as the internal control (Additional file 4: Figure S4).

A marked increase in the expression of *PAP5* at 6 h.p.i was observed in wild-type plants (Figure 4B). However, this difference did not prolong to 24 and 48 h.p.i. We did not observe induction of *PAP5* in mock infected or *Pst* DC3000 inoculated *pap5-1* plants (Figure 4B)*.* The expression profile of *PAP5* was further verified from the comprehensive microarray site http://bar.utoronto.ca/ using Arabidopsis eFP Browser (Additional file 5: Figure S5) [35]. Although, *PAP5* was strongly induced only at 6 h.p.i, our results suggest that this level of *PAP5* is required for maintaining resistance against virulent *Pst* DC3000.

**Figure 5:**
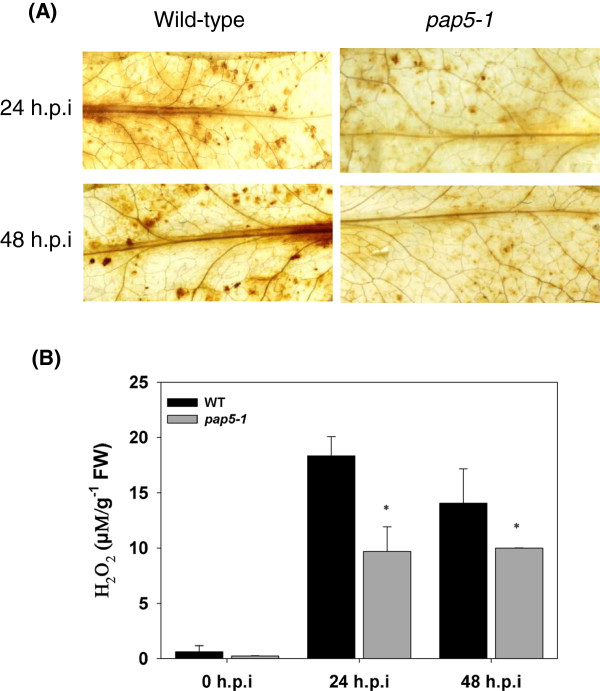
***pap5-1 *plants accumulate reduced H**_**2 **_**O**_**2 **_**in response to *Pst *DC3000 infection. A**, Histochemical detection of H_2_O_2_ accumulation using DAB staining. Wild-type and *pap5-1* leaves were excised following *Pst* DC3000 infection and stained with DAB for hydrogen peroxide. **B**, Quantification of H_2_O_2_ following *Pst* DC3000 infection. The bars represent mean and SD of H_2_O_2_ accumulation. Asterisks represent significant difference in H_2_O_2_ production compared to wild type (Student’s *t*-test; *P* < 0.05).

To further explore the mechanism of enhanced susceptibility, we studied hydrogen peroxide (H_2_O_2_) accumulation using 3-3’-Diaminobenzidine (DAB) staining. As shown in Figure 5A, accumulation of H_2_O_2_ in response to *Pst* DC3000 was reduced in *pap5-1* leaves at 24 and 48 h.p.i. In contrast, there was an accumulation of H_2_O_2_ in the wild-type plants. The H_2_O_2_ concentration was quantified in leaf tissues following *Pst* DC3000 infection. The wild-type plants accumulated a higher concentration of H_2_O_2_ in response to *Pst* DC3000 inoculation as compared to *pap5-1* plants (Figure 5B).

### Resistance to *Botrytis cinerea* is affected in *pap5* plants

**Figure 6:**
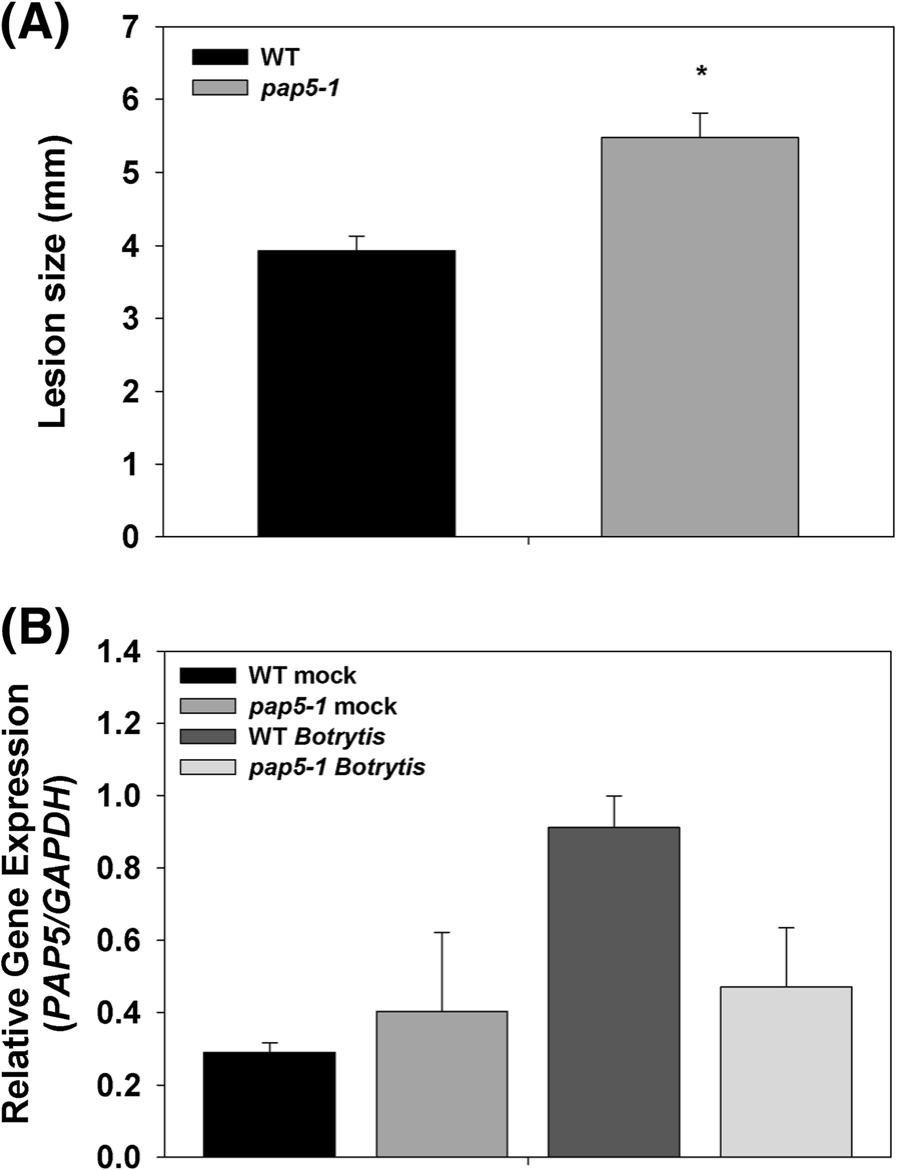
**Lesion development and induction of *PAP5 *following *Botrytis cinerea *infection. A**, Size of lesion in wild type and *pap5-1* plants inoculated after *B. cinerea* infection. Leaves were inoculated by placing 5 μl of the *B. cinerea* spore suspension (1 × 10^5^/ml) on either side of the mid vein and the lesion size was measured after 3 days. The bars represent mean and SD of 20 individual lesions. Asterisks represent significant difference in lesion size compared to wild-type (Student’s *t*-test; *P* < 0.05). **B**, *PAP5* expression in response to *B. cinerea* infection. Leaf tissues were harvested 48 h.p.i for RNA extraction. Transcript levels of *PAP5* were normalized to the expression of *GAPDH* in the same samples. The transcript levels were expressed relative to the normalized transcript levels of infected wild-type plants.

Having demonstrated the enhanced susceptibility of *pap5-1* plants to the hemi-biotrophic pathogen *Pst* DC3000, we next tested the level of resistance of *pap5-1* plants to the necrotrophic pathogen *Botrytis cinerea*. Four week old plants were inoculated with spore suspension of *B. cinerea* and lesion size was measured three days later. As shown in Figure 6A, *pap5-1* plants developed a significantly larger lesion (5.4 ± 0.3 mm) than the wild-type (3.9 ± 0.2 mm). The greater lesion size on *pap5-1* plants in response to *B. cinerea* infection, suggests the role of *PAP5* are important in limiting fungal growth.

**Figure 7:**
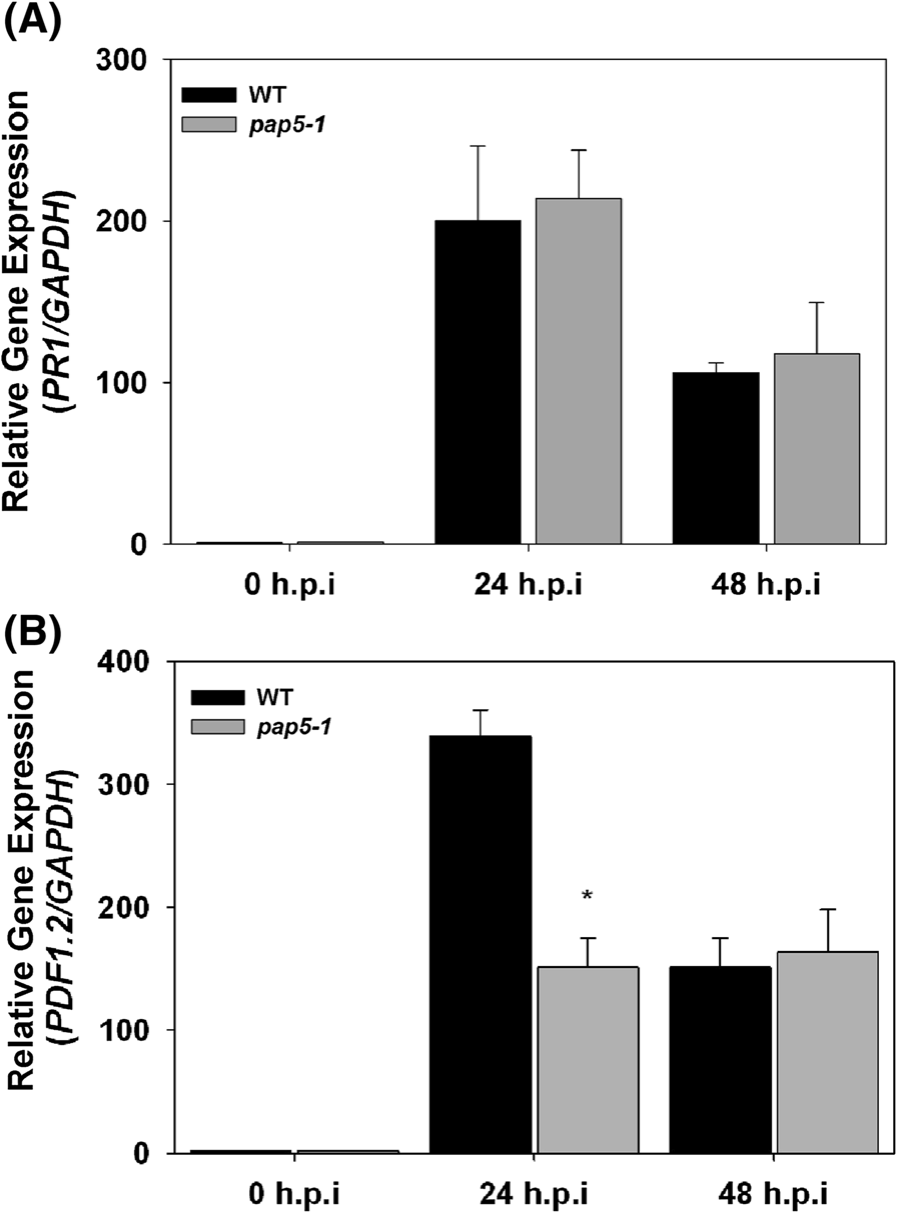
**Induction of *PR1 *and *PDF1.2 *in response to *Botrytis cinerea*. A**, Expression *PR1* in response to *B. cinerea* infection. **B**, expression of *PDF1.2* in response to *B. cinerea* infection. Plants were spray inoculated with spore suspension of *B. cinerea* (1 × 10^5^) and leaf tissues were harvested for total RNA extraction. Transcript levels of *PR1* and *PDF1.2* was normalized to the expression of *GAPDH* in the same samples and expressed relative to the normalized transcript levels of mock infected wild-type plants. The bars represent the mean and standard deviation from two independent experiments. Asterisks indicate significant difference in transcript levels compared to wild-type (Students *t*-test; *P* < 0.05). Induction of *PR1* and *PDF1.2* in response to *Botrytis cinerea* infection.

To identify the role of *PAP5* in the resistance against *B. cinerea,* we assessed the transcript abundance of *PR1* and *PDF1.2*. As shown in Figure 7A, *B. cinerea* strongly induced the expression of *PR1* in both wild-type and *pap5*. In contrast, the level of the *PDF1.2* transcript at 24 h.p.i was only half of that observed in wild-type plants (Figure 7B). By 48 h.p.i., however, the transcript levels of *PDF1.2* were similar in both wild-type and *pap5-1* plants. Similarly, we did not observe any significant differences in *PAP5* transcripts with *B. cinerea* infection (Figure 6B).

### Responses to exogenous application of BTH, a salicylic acid analog and methyl jasmonate (MJ) is unaffected in *pap5* plants

**Figure 8:**
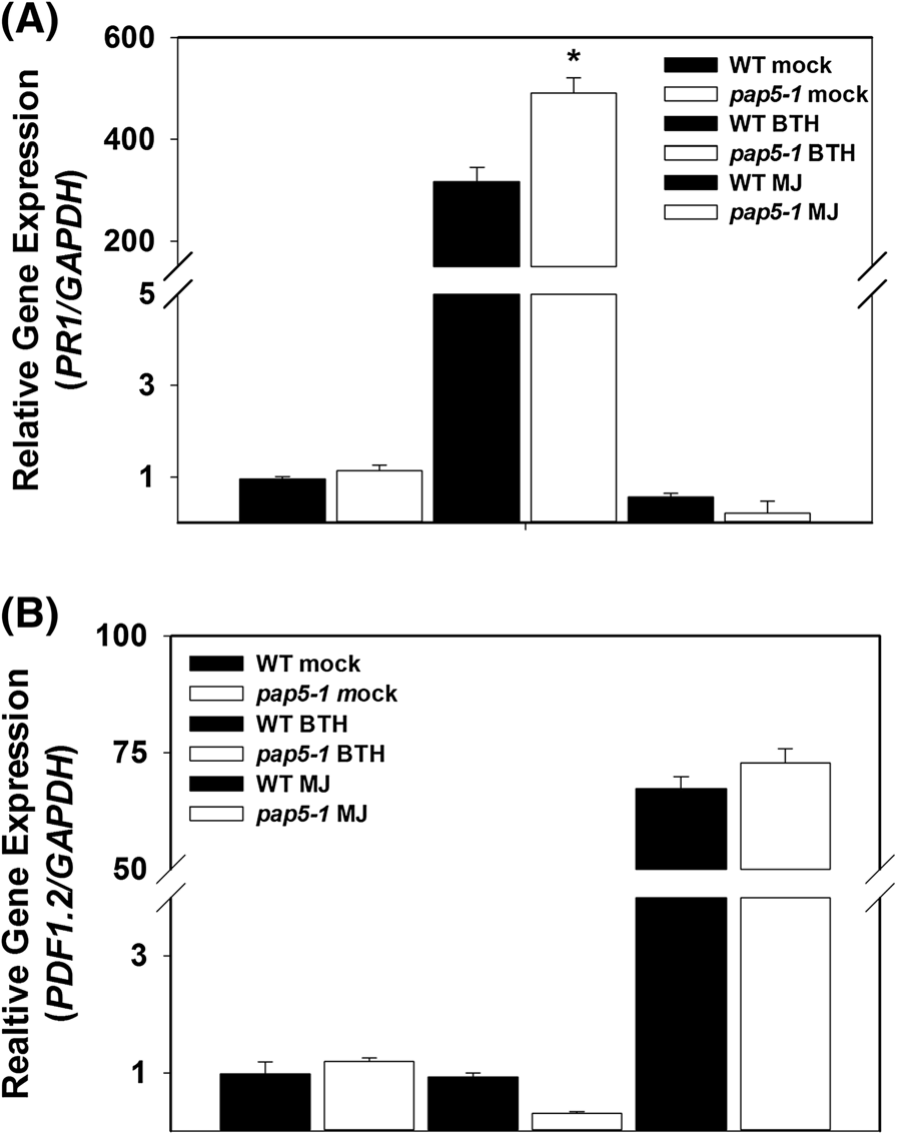
**Induction of *PR1 *and *PDF1.2 *following exogenous application of benzothiozidole and methyl jasmonate. A**, Expression *PR1* in response to benzothiozidole (BTH) treatment. **B**, expression of *PDF1.2* in response to methyl jasmonate treatment. Plants were spray treated with either or 0.06% of Actigard® (Active ingradient: 50% w/w benzothiozidole) or 50 μM methyl jasmonate. Leaf tissues were harvested after 24 of spraying for total RNA extraction. Transcript levels of *PDF1.2* and *PR1* were normalized to the expression of *GAPDH* in the same samples and expressed relative to the normalized transcript levels of mock treated wild-type plants. The bars represent the mean and standard deviation from two independent experiments. Asterisks indicate significant difference in transcript levels compared to wild-type (Students *t*-test; *P* < 0.05).

Since *pap5-1* plants exhibited enhanced susceptibility to *Pst* DC3000 and *B. cinerea,* we investigated the role of *PAP5* in responses to BTH and MJ. Exogenous application of BTH induced higher levels of *PR1* in wild-type and *pap5-1* (Figure 8A). We also observed a slightly higher increase in the expression *PR1* in *pap5-1* plants 24 h after BTH treatment. Similarly, application of MJ strongly induced the expression of *PDF1*.*2* in both wild-type and *pap5-1* plants. We did not observe significant differences in expression of *PDF1*.*2* between wild-type and *pap5-1* plants following application of MJ (Figure 8B). Application of BTH and JA induced expression of *PR1* and *PDF1.2*, respectively, indicative of an intact JA signaling pathway in *pap5* plants. Based on these experiments it was clear that *pap5-1* plant was not defective in responding to exogenously applied BTH or MJ.

## Discussion

In this study, we demonstrated the role of *PAP5*, a phosphate responsive gene, and its requirement in maintaining basal disease resistance against virulent *Pst* DC3000. In previous studies *PAP5* transcripts were not detectable under phosphate starvation [36]. Unlike *PAP12* and *PAP26, PAP5* is not abundantly expressed under normal phosphate starvation conditions. Our results revealed that *PAP5* is expressed only under prolonged Pi starvation (Figure 2C). Mutation in *PAP26* has been shown to impair growth and increase anthocyanin accumulation in response to Pi starvation [37]. Despite the loss of *PAP5* expression, mutant plants did not show discrete phenotypic differences from that of wild-type plants. Both wild-type and *pap5-1* plants exhibited an increased root/shoot ratio under Pi starvation (data not shown). This finding also indicates that *PAP5* does not play a major role in Pi acquisition and is more likely to regulate other functions. The Arabidopsis genome contains 29 *PAP* encoding genes [33] and this may lead to functional redundancy. This study suggests that the loss of *PAP5* resulted in impairment of defense responsive genes in response to *Pst* DC3000 infection. Further, it appears that other *PAP* genes does not compensate for the loss of *PAP5* function in response to pathogen attack*.*

Genetic analyses of Arabidopsis mutants have revealed many key regulatory genes in plant defense responses. Enhanced disease susceptibility mutants including *eds5*, *pad4*, *npr1* and *sid2* have previously been reported to exhibit enhanced susceptibility and compromised defense responses to both virulent and avirulent isolates of *Pst* DC3000 [19,20,38]. It is also evident that most bacterial pathogens including *Pst* DC3000 are inoculated by pressure-infiltration to study plant-bacterial interactions. Although pressure-infiltration is the most commonly used inoculation method, these inoculation procedures may prevent early innate immune responses such as flagellin perception (FLS2 mediated resistance) [39] and stomatal closure [40]. Also FLS2 mediated resistance was effective only when *Pst* DC3000 was sprayed on the leaf surface and not when bacteria was infiltrated in to leaves [39]. Hence, to mimic natural infection and to focus on the early defense responses we sprayed plants with suspension of *Pst* DC3000 containing 10^8^ c.f.u ml^–l^. We also observed that plants sprayed with 10^3^ and 10^5^ cells/ml^-l^ developed reduced symptoms compared to plants sprayed with 10^8^ cells (data not shown). Similar bacterial titers have been previously used for plant-bacterial interaction studies [39,40].

We observed that the expression of *PR1* was slightly induced in *pap5-1* plants following *Pst* DC3000 infection, however the relative transcript level of *PR1* was several fold lower compared to wild-type (Figure 3). The *PR1* transcripts at 48 h.p.i were slightly lower compared to wild-type (Figure 3). Similar variability has been observed in MPK6 silenced plants that were susceptible to *Pst* DC3000 [41]. We observed that *PAP5* was strongly induced in the early stages of infection (6 h.p.i). This induction was transient as no difference was observed at 24 and 48 h.p.i. One possible explanation of this observation is that the level of PAP5 induced during the early stages (6 h.p.i) of infection could be sufficient to dephosphorylate signaling proteins required for activation of defense responses downstream of *PAP5*. Thus, it is also possible that *PAP5* might be involved in early responses to pathogens similar to glutotione s-transferse (*GST6*) and glucosyltransferase [42]. Moreover, members of the PAP family have been known to exhibit peroxidase activity in addition to Pi acquisition and recycling [24,43]. Although, the role of *PAP5* with regard to peroxidase activity has not been established, we hypothesize that the *PAP5* might mediate generation of reactive oxygen species (ROS) during *Pst* DC3000 infection. ROS was initially proposed to be mediate plant defense response especially, during an incompatible interaction [44]. Virulent pathogens, capable of evading pathogen recognition are also known to induce ROS production at latter stages of infection to lower levels [1].

We also identified the importance in *PAP5* in limiting the growth of the necrotrophic fungus, *B. cinerea* at the site of infection. The expression of *PDF1.2* was strongly suppressed in *pap5-1* plants at 24 h.p.i resulting in an increase in lesion size. There were no differences in *PDF1.2* transcripts between *pap5* and wild-type plants at 48 h.p.i. Similarly, *eds4-1* plants have been reported to exhibit enhanced susceptibility to *B. cinerea* despite comparable expression of *PR1* and *PDF1.2* transcripts [45]. These results also suggest that defense responsive genes other than *PR1* and *PDF1.2* are required to mount wild-type levels of resistance against *B. cinerea*. SA synthesized in response to *B. cinerea* infection has reported to be derived via phenylalanine ammonia lyase (*PAL*) and not via isochorismate synthase (*ICS*) [46]. Since *pap5-1* plants induced comparable levels of *PR1* to wild-type plants following *B. cinerea* infection, it is possible that the effect of *PAP5* is restricted to SA derived via *ICS* and not via *PAL*.

Application of BTH and MJ in wild-type and *pap5* plants induced expression of *PR1* and *PDF1.2,* respectively (Figure 8A and 8B). These results also suggest that *PAP5* is not required for expression of SA dependent *PR1* expression. *PR1* expression in *pap5-1* plants appeared to be slightly higher than wild-type plants after of BTH treatment (Figure 8A). This slight increase in *PR1* expression and its significance is unclear. Similarly, application of SA on *pad4* plants showed a slight increase in *PR1* expression [47]. Application of MJ induced the expression of *PDF1*.*2,* indicating the regulatory function of *PAP5* to be upstream of SA and JA.

**Figure 9:**
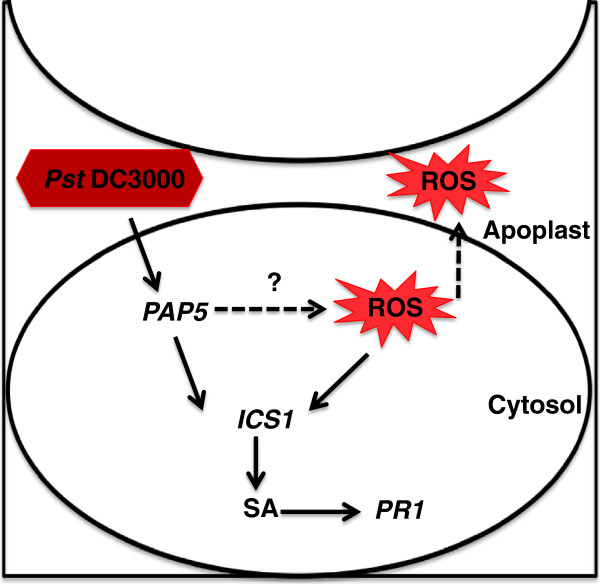
**Model for role of PAP5 during *Pst *DC3000 infection.** When plants are infected with virulent *Pst* DC3000, PAP5 is required for activation of defense responsive genes including *PR1* and *ICS1*. Recognition of *Pst* DC3000, induce expression of *PAP5* only during the early stages of infection (6 h) and triggers ROS synthesis which subsequently activates other defense related signals down stream for complete resistance.

Although, most plant PAPs are primarily associated with Pi absorption and recycling, PAPs induced under Pi starvation are also known to exhibit peroxidase activity similar to mammalian PAPs [24,48]. All mammalian PAPs characterized exist as monomers of ~35 kDa (Low Molecular Weight, LMW), while plants encode a relatively large family of High Molecular Weight (HMW) homodimeric and oligomeric PAPs (~45-74 kDa). However, a recent study has identified mammalian-like low molecular weight PAP (~34 kDa) from roots of Pi starved bean plants [32]. Moreover, the LMW, 35 kDa plant PAPs are reported to be closely related to the 35 kDa mammalian PAPs than to the large plant PAPs [49]. Thus, from our results we hypothesize that PAP5 could play a role in both Pi acquisition and in microbial killing during pathogenesis (Figure 9).

## Conclusion

We identified the requirement of *PAP5* for maintaining basal defense responses against virulent *Pst* DC3000, suggesting a role for *PAP5* in pathogen triggered immunity (PTI). We further demonstrated that *PAP5* acts upstream of SA to affect the expression of *PR1,* and levels of *PAP5* do not affect BTH and JA perception. Further analysis on *pap5* plants is likely to reveal novel components of signal transduction pathways that regulate defense responsive genes.

## Methods

### Biological materials and growth conditions

Arabidopsis thaliana (L.) Heynh, ecotype Columbia (Col-0) seed was purchased from Lehle seeds (Round Rock, TX, USA) and T-DNA insertion mutant lines were obtained from Arabidopsis Biological Resource Center (Columbus, OH, USA). Seeds were surface sterilized with NaOCl 2% (v/v), rinsed five times with sterile water and stratified at 4°C for 3 days. Seeds were planted either in Jiffy peat pellets (Halifax seeds, Canada) or on plates with 0.5X MS media [50]. Plants were grown at 22 ± 2°C with a photoperiod of 16 h light at 125 μmol m^-2^ s^-1^ and 8 h dark cycle.

Virulent *Pseudomonas syringae* pv. *tomato* DC3000 (*Pst* DC3000) was kindly gifted by Dr. Diane Cuppels, Agriculture and Agri Food Canada (AAFC), ON, Canada. *Pseudomonas syringae* strain was maintained on King’s medium B supplemented with rifampicin (50 μg ml^-l^). *Botrytis cinerea* was cultured on modified King’s medium B (10 g peptone, 1.5 g potassium phosphate monobasic, 15 g dextrose, pH 5.5, 5 ml of 1 M MgSO4/l).

For plant treatment, Benzothiozidole (Actigard®; active ingradient 50% w/v BTH) was a gift from Syngenta Corp., USA. Methyl jasmonate and other microbiological media were purchased from Sigma Aldrich, Oakville, Canada.

### Mutant screening and pathogen inoculation

Genetic screen was performed on 4 to 5 week old plants by spray inoculation with bacterial suspension of virulent *Pst* DC3000. Plant inoculation and bacterial growth in plant apoplast was determined as described by [39]. In brief, strains of virulent *Pseudomonas syringae* pv. *tomato* DC3000 (*Pst* DC3000) was cultured in King’s medium B supplemented with rifampicin (50 μg ml^-l^) at 28°C until OD_600_ of 0.8. Bacterial cells were collected by centrifugation and resuspended in water containing 0.02% Silwet L-77 (Lehle seeds, USA) to a final concentration of 10^8^ c.f.u ml ^-l^. Plants (4-5 weeks old) were spray inoculated and kept under high humidity for disease development. Leaves were excised (8-10 replicates) from different infected plants and were surface sterilized with ethanol (75% v/v). Four to five samples were made by pooling 2 leaf discs (0.5 cm^2^) and the samples were ground in sterile water with microfuge tube pestle. The ground tissues were serially diluted and plated on King’s B medium containing rifampicin (50 μg/ml). The plates were incubated at 28°C and colonies were counter after 48 hours. For *Pst* DC3000 induced gene expression, plants were spray inoculated with bacterial suspension (10^8^ c.f.u ml ^–l^) and leaf tissues were frozen in liquid nitrogen at the time points indicated.

For *Botrytis cinerea* (*Bcr*) inoculation, spore suspension (1 × 10^5^ conidia mL^-1^) was prepared in potato dextrose broth (PDB) as described by [45]. Four to five week old plants were inoculated by placing 5 μl of the spore suspension on either side of the mid vein of fully expanded leaves. Inoculated plants were covered with a transparent plastic dome to maintain high humidity for disease development. For all gene expression analysis, leaf tissues were harvested from four individual plants for each biological replicate and were snap frozen in liquid nitrogen for RNA extraction.

Benzothiozidole (BTH) and methyl jasmonate (MJ) treatments were performed by spraying 4-5 weeks old plants with solutions containing 0.06% w/v Actigard® (Active ingradient: 50% w/v BTH) or 50 μM methyl jasmonate (MJ) with 0.02% Silwet L-77.

### Confirmation of T-DNA insertion

T-DNA insertion and homozygosity of mutant line salk_126152 was confirmed by PCR as described by [51] using *AtPAP5* gene specific primers generated from SALK T-DNA verification primer design tool LP 5’-TTCACGGTTTTGTTGTTAGACG-3’, RP 5’-TCGTTGAAAACTACACTCGATTTAAC-3’ and left border primer LBb1.3 5’-ATTTTGCCGATTTCGGAAC-3’.

### Phosphate starvation

Sterile, stratified seeds (20-25 per jar) were dispensed in 50 ml of liquid 0.5X MS medium containing Pi (1.25 mM) or with reduced Pi (0.25 mM). The seedlings were grown under constant shaking (85 rpm) at 22 ± 2°C under continuous illumination at 100 μmol m^-2^ s^-1^. After 9 days the seedlings were rinsed thrice with sterile distilled water and transferred to 0.5X MS medium containing + Pi (1.25 mM) or –Pi (0 mM) [52]. Plants were harvested after 11 days for RNA extraction. Whenever Pi was reduced from growth medium, equivalent amounts of sulphate salts were added to maintain the concentration of conjugate cations.

### RNA extraction and quantitative Real-time PCR

Total RNA was extracted from frozen tissues using monophasic extraction method [53]. Reverse Transcription was performed with 2 μg of total RNA using Quantiscript RTase (Qiagen, ON, Canada). Relative transcript levels were assayed by Real-Time PCR using gene specific primers (Table 1) on StepOnePlus Real-Time PCR system (Applied Biosystems, ON, Canada) using SYBR Green reagent (Applied Biosystems, ON, Canada). To determine the relative expression levels, the amount of target gene was normalized over the abundance of constitutive *Glyceraldehyde 3-phosphate dehydrogenase* (*GAPDH*) or *Actin* as endogenous control. Primers were generated using the Roche Universal Probe Library assay design center.

**Table 1:** Primer sequences used in RT-qPCR experiments

**Gene**	**Locus**	**Primer sequences (5’-3’)**
*GAPDH*	At1g13440	TTGGTGACAACAGGTCAAGCA
		AAACTTGTCGCTCAATGCAATC
*ICS1*	At1g74710	GCGTCGTTCGGTTACAGG
		ACAGCGAGGCTGAATATCAT
*PAP5*	At1g52940	AACAGGTCGCTCCACTAGACA
		TGGTTAGAGGCATATGTTTGTCC
*PDF1*.2	At5g44420	GTTCTCTTTGCTGCTTTCGAC
		GCAAACCCCTGACCATGT
*PR1*	At2g14610	TGATCCTCGTGGGAATTATGT
		TGCATGATCACATCATTACTTCAT

### DAB staining

To visualize H_2_O_2_ production *in situ*, plants were inoculated with suspension of *Pst* DC3000 as described in earlier section. Leaves were excised at 24 and 48 h.p.i and stained with 3-3 Diaminobenzidine (DAB) as described by [54]. Excised leaved were placed in DAB (1 mg/ml) solution for 8-12 hours and the tissues were soaked in ethanol (95%, v/v) to remove chlorophyll. For H_2_O_2_ quantification, the excised leaf tissues were frozen and ground with liquid nitrogen. To 50 mg of ground frozen tissue 500 μl of phosphate buffer (50 mM, sodium phosphate, pH-7.4) was added. The samples were centrifuged and 50 μl of the aliquot was used for H_2_O_2_ quantification using an Amplex red hydrogen peroxide/peroxidase assay kit (Molecular Probes, Life Technologies, Canada).

## Competing interests

The authors declare that they have no competing interests.

## Authors’ contributions

BP conceived the concept of genetic screening and designed all experiments. SR carried out all the experiments and prepared the manuscript. SLS critically evaluated all the experiments and significantly contributed to the manuscript preparation. BB helped with sequencing and contributed to evaluate the manuscript. All authors read and approved the final manuscript.

## Supplementary Material

Additional file 1: Figure S1 Enhanced susceptibility of *pap5-2* to *Pst* DC3000. A, Phenotype of *pap5-2* plants exhibiting extensive chlorosis. Plants were spray inoculated with 10^8^ c.f.u ml^–l^ and photographed after 5 days of infection. B, Growth of virulent *Pst* DC3000 in wild type (Col-0) and *pap5-2* mutant leaves. Plants were spray inoculated with *Pst* DC3000 (10^8^ c.f.u ml^–l^) and bacterial growth in plant apoplast was determined. The bars represent the mean and standard deviation from values of six to eight replicate samples and the experiment was repeated two times with similar results. An asterisk indicates significant increase in *Pst* DC3000 growth compared to wild-type (Student’s *t*-test; *P* < 0.05). Click here for file

Additional file 2: Figure S2 Expression profile of *PAP5* (array element 261341_s_at) in comparison to *PR1* (array element 266385_at) from Genevestigator Expression Data. Click here for file

Additional file 3: Figure S3 Validation of T-DNA insertion in *pap5-2* mutant plants. A, Schematic representation of *AtPAP5* (At1G52940); white boxes and solid lines represent exons and introns. T-DNA insertion is represented with a grey arrow and the solid arrows represent the primers used for genotyping and quantitative RT-qPCR. B, Location of the T-DNA insertion and homozygosity of *pap5-2* was confirmed by PCR using the gDNA from wild-type and *pap5-2* plants (M, 100 bp marker). A 30 cycle PCR reactions was performed with the primer pairs indicated. C, Relative expression of *PAP5* transcripts in response to Pi starvation; Total RNA was extracted from wild-type and *pap5* plants as described in materials and methods. Transcript levels of *PAP5* was normalized to the expression of *GAPDH* in the same samples and expressed relative to the normalized transcript levels of Pi supplemented wild-type plants. The bars represent the mean and standard deviation from two independent experiments. Asterisks represents data sets significantly different from the wild-type data sets (*P* < 0.05 using one-tailed Student’s *t*-test). Click here for file

Additional file 4: Figure S4 Expression of defense related genes in wild-type and *pap5-1* mutant plants after *Pst* DC3000 infection. Transcript levels of *PR1*, *ICS1*, *PDF1.2* and *PAP5* in wild-type and *pap5-1* plants were quantified after spray inoculation with virulent *Pst* DC3000 (10^8^ c.f.u ml^–l^) was determined. Total RNA was extracted from leaf tissues harvested 24 h.p.i*.* Transcript levels were normalized to the expression of *Actin* in the same samples. The transcript levels were expressed relative to the normalized transcript levels of mock infected wild-type plants. The bars represent the mean and standard deviation. Significant differences (P < 0.05) are indicated by different letters. Click here for file

Additional file 5: Figure S5. Expression profile of *PAP5* (At1g52940) from the Arabidopsis eFP Browser. Click here for file
