# Supplementary figures and images for: Purple Acid Phosphatase5 is required for maintaining basal resistance against Pseudomonas syringae in Arabidopsis
Source: BMC Plant Biol. 2013 Jul 29;13:107. doi: 10.1186/1471-2229-13-107 (PMC3751912; doi:10.1186/1471-2229-13-107)

## Slide 1
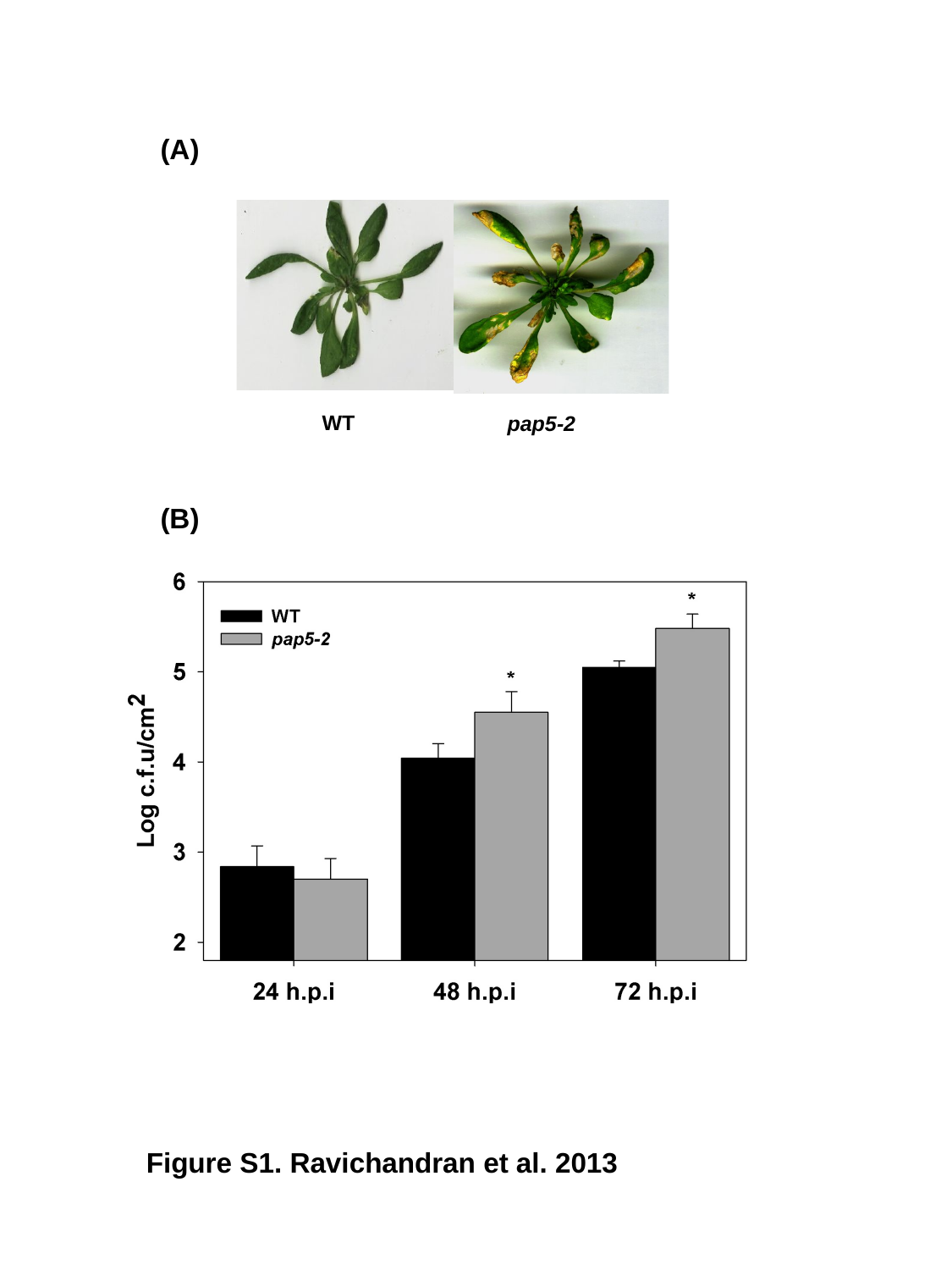

(A)
WT
pap5-2
(B)
Figure S1. Ravichandran et al. 2013

Supplement: Additional file 1: Figure S1 — Enhanced susceptibility of pap5-2 to Pst DC3000. A, Phenotype of pap5-2 plants exhibiting extensive chlorosis. Plants were spray inoculated with 108 c.f.u ml–l and photographed after 5 days of infection. B, Growth of virulent Pst DC3000 in wild type (Col-0) and pap5-2 mutant leaves. Plants were spray inoculated with Pst DC3000 (108 c.f.u ml–l) and bacterial growth in plant apoplast was determined. The bars represent the mean and standard deviation from values of six to eight replicate samples and the experiment was repeated two times with similar results. An asterisk indicates significant increase in Pst DC3000 growth compared to wild-type (Student’s t-test; P < 0.05). [file 1471-2229-13-107-S1.pptx]

## Slide 1
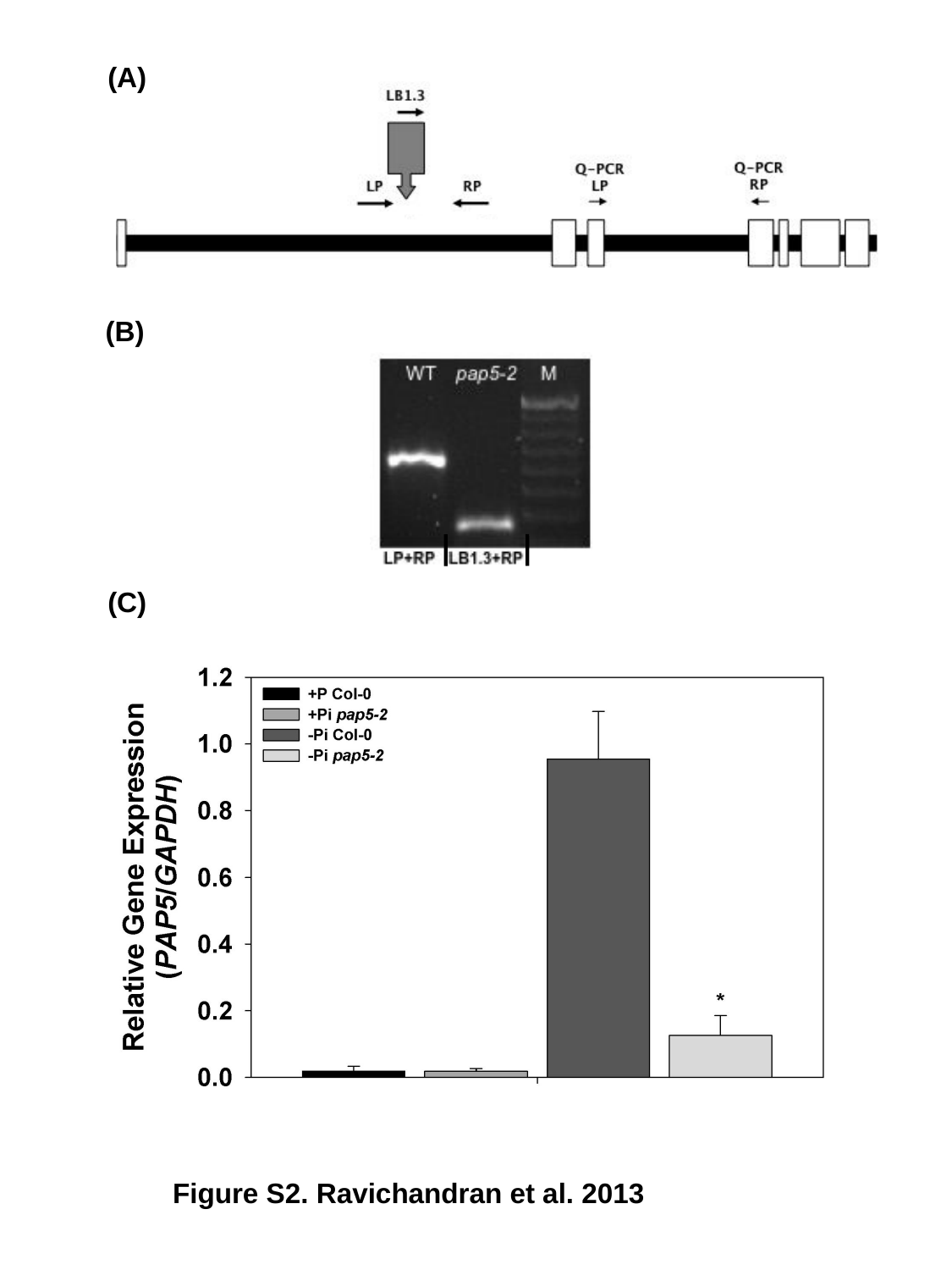

(A)
(B)
(C)
Figure S2. Ravichandran et al. 2013

Supplement: Additional file 2: Figure S2 — Expression profile of PAP5 (array element 261341_s_at) in comparison to PR1 (array element 266385_at) from Genevestigator Expression Data. [file 1471-2229-13-107-S2.ppt]

## Slide 1
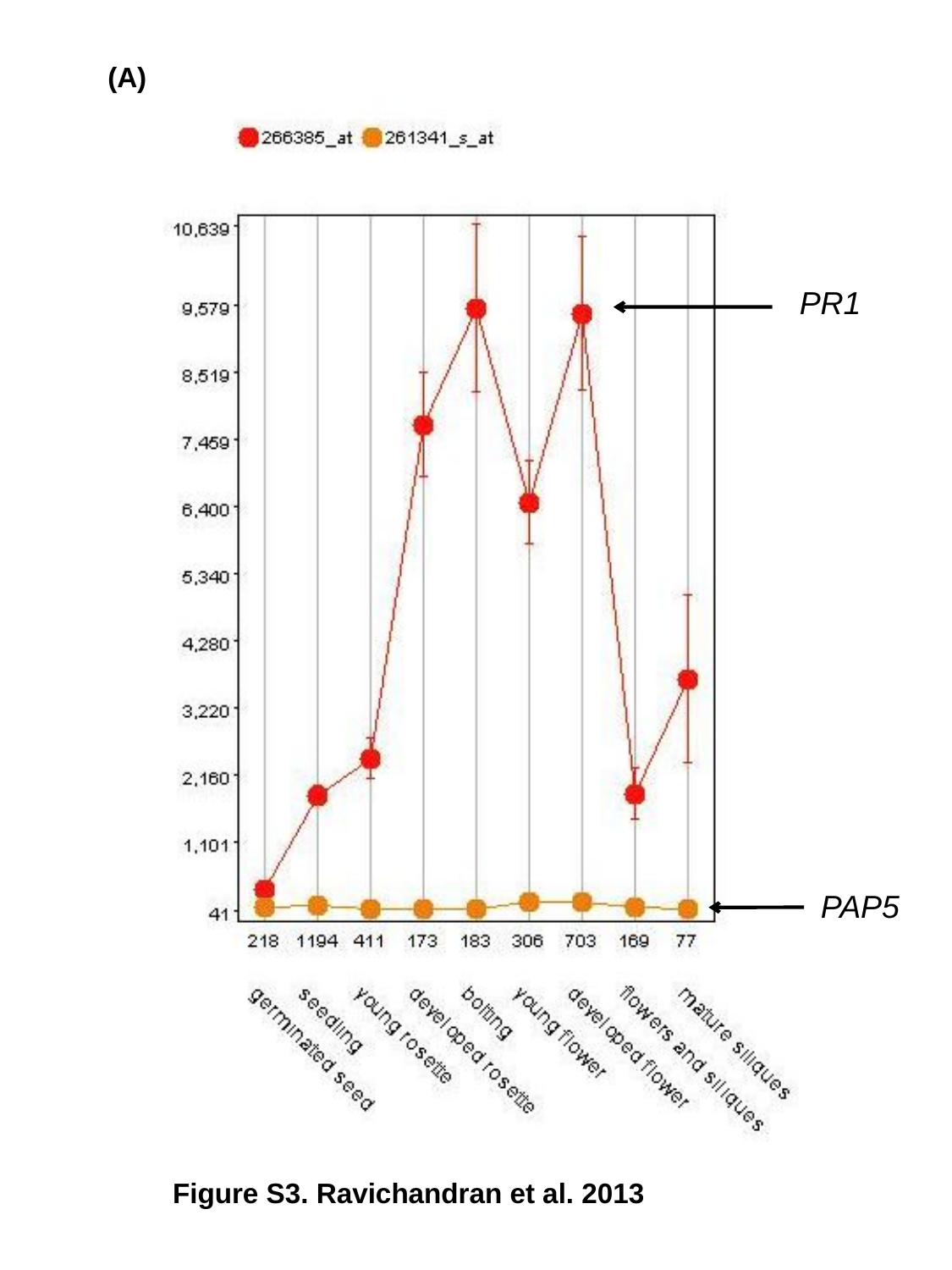

(A)
PR1
PAP5
Figure S3. Ravichandran et al. 2013

Supplement: Additional file 3: Figure S3 — Validation of T-DNA insertion in pap5-2 mutant plants. A, Schematic representation of AtPAP5 (At1G52940); white boxes and solid lines represent exons and introns. T-DNA insertion is represented with a grey arrow and the solid arrows represent the primers used for genotyping and quantitative RT-qPCR. B, Location of the T-DNA insertion and homozygosity of pap5-2 was confirmed by PCR using the gDNA from wild-type and pap5-2 plants (M, 100 bp marker). A 30 cycle PCR reactions was performed with the primer pairs indicated. C, Relative expression of PAP5 transcripts in response to Pi starvation; Total RNA was extracted from wild-type and pap5 plants as described in materials and methods. Transcript levels of PAP5 was normalized to the expression of GAPDH in the same samples and expressed relative to the normalized transcript levels of Pi supplemented wild-type plants. The bars represent the mean and standard deviation from two independent experiments. Asterisks represents data sets significantly different from the wild-type data sets (P < 0.05 using one-tailed Student’s t-test). [file 1471-2229-13-107-S3.ppt]

## Slide 1
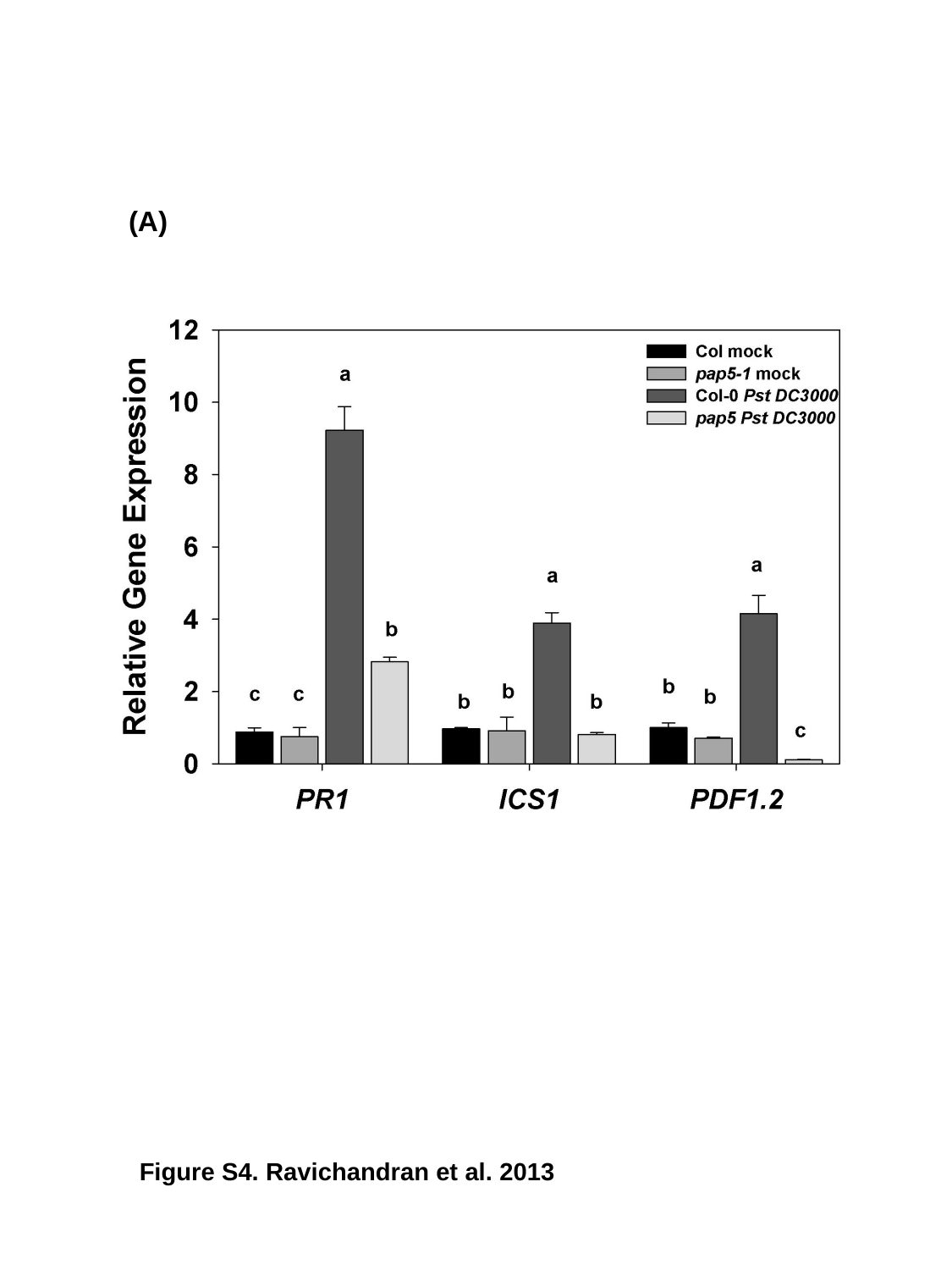

(A)
Figure S4. Ravichandran et al. 2013

Supplement: Additional file 4: Figure S4 — Expression of defense related genes in wild-type and pap5-1 mutant plants after Pst DC3000 infection. Transcript levels of PR1, ICS1, PDF1.2 and PAP5 in wild-type and pap5-1 plants were quantified after spray inoculation with virulent Pst DC3000 (108 c.f.u ml–l) was determined. Total RNA was extracted from leaf tissues harvested 24 h.p.i. Transcript levels were normalized to the expression of Actin in the same samples. The transcript levels were expressed relative to the normalized transcript levels of mock infected wild-type plants. The bars represent the mean and standard deviation. Significant differences (P < 0.05) are indicated by different letters. [file 1471-2229-13-107-S4.ppt]

## Slide 1
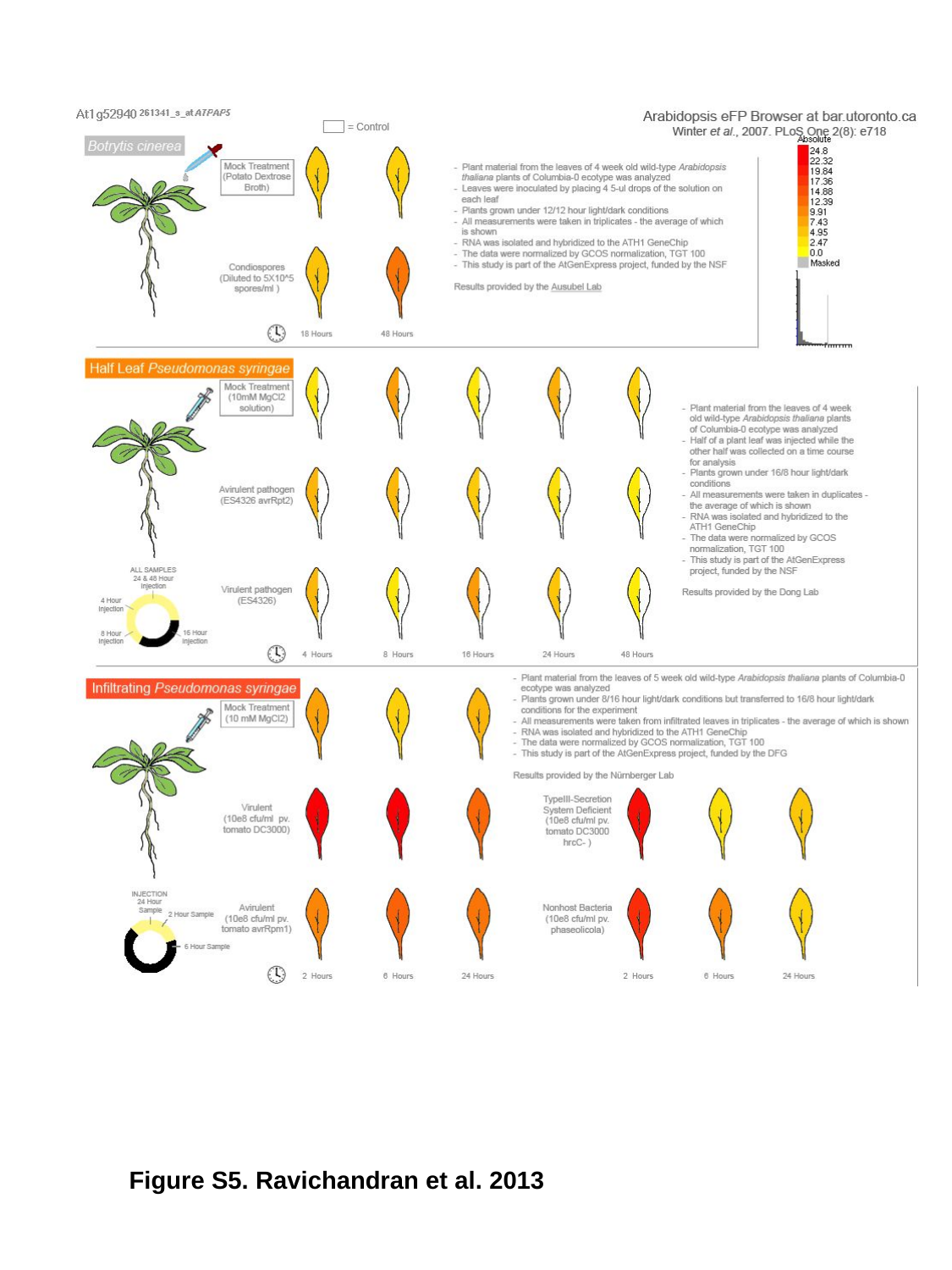

Figure S5. Ravichandran et al. 2013

Supplement: Additional file 5: Figure S5. — Expression profile of PAP5 (At1g52940) from the Arabidopsis eFP Browser. [file 1471-2229-13-107-S5.pptx]
